# Rage and Ruin

**Published:** 1880-03

**Authors:** 


### RAGE AND RUIN.

Some one has said, that every furious burst of passion shortens a man's life a year. If it only shortened his own life, the world would not be a great loser ; but unfortunately, passionate people keep all around them in hot water ; their very presence, without a word being said, generates an evil atmosphere.

